# Enhancing Participation in Alzheimer’s Research: A Comparative Study of Community Health Worker -Led and Digital Recruitment Strategies in Hispanic/Latino Communities

**DOI:** 10.1007/s40615-026-02886-3

**Published:** 2026-03-19

**Authors:** Yelba Castellon-Lopez, Leslie Aguilar-Hernandez, Iris Y. Guzman-Ruiz, Susana Fernandez de Cordova, Maria del Carmen Rosales, Javier Neira Salazar, Maria Mora Pinzon

**Affiliations:** 1 Department of Biomedical Sciences, Cedars-Sinai Medical Center, Cancer Research Center for Health Equity, Samuel Oschin Comprehensive Cancer Institute, Los Angeles, CA, USA; 2 Division of Geriatrics and Gerontology, Department of Medicine, School of Medicine and Public Health, Madison, University of WI–Madison, Madison, WI, USA; 3 Wisconsin Alzheimer’s Institute, University of WI–Madison, Madison, WI, USA

**Keywords:** Community health worker, Health promotors, Underrepresented populations, Community engagement, Online recruitment, Recruitment strategies

## Abstract

**Background:**

Digital recruitment can expand geographical reach and reduce recruitment timelines. Community health worker (CHW)-led recruitment has shown to build trust, increase knowledge, and link patients to healthcare services and research participation. However, few studies quantify how recruitment strategies relate to research outcomes.

**Objective:**

To explore associations between recruitment strategies and participant demographics and memory disorder knowledge using a survey with a sample of Hispanic/Latino participants.

**Methods:**

We conducted a cross-sectional comparative analysis of recruitment and survey responses in a survey study targeting U.S. Hispanic/Latino individuals using both digital and CHW-led recruitment strategies. We compare participant demographics and knowledge of memory disorders using chi-squared tests for categorical variables and t-tests for continuous variables.

**Results:**

We recruited 834 participants across both strategies. Recruitment strategy was associated with educational background, basic Alzheimer’s disease (AD) knowledge, and misconceptions. In the CHW-recruited group, a higher proportion had less than a high school diploma (29.41% vs. 5.13% in the digital group (*p* < .001). CHW-recruited participants also scored lower on the Basic Knowledge of AD survey (19.89 vs. 21.85, *p* < 0.001) and held more misconceptions, such as believing AD is a normal part of aging (*M* = 2.12, *SD* = 1.04, *p* < .001).

**Conclusions:**

Findings from this study suggest that CHW-led recruitment might be more effective at engaging a greater proportion of individuals from traditionally underrepresented groups, such as those of Hispanic/Latino heritage reporting lower educational attainment, and Spanish-language preference, compared to digital-led recruitment. These insights underscore the importance of diversifying recruitment strategies to meet the specific needs of underrepresented demographic groups and can inform experimental research to strengthen recruitment science.

## Introduction

Recruitment is a critical component of clinical and translational research to ensure diversity and representativeness of study populations. Nevertheless, specific demographic groups, such as the Hispanic/Latino community in the United States (U.S.), remain persistently underrepresented in research participation. [1, 2]. Historically, this trend has been attributed to factors such as mistrust in the medical system, language barriers, and various socioeconomic and cultural challenges that discourage U.S. Hispanic/Latino individuals from engaging with the healthcare system and participating in clinical research studies [3-5]. The lack of diverse participation in research limits the generalizability of study findings, posing the risk that interventions and treatments may be less effective or even harmful for underrepresented groups [6-8]. Similarly, survey studies that do not adequately represent all groups can lead to a limited understanding of the health needs of diverse groups, who often carry a disproportionate burden of risk, thereby contributing to health disparities.

Given the scientific challenges posed by underrepresentation, more research is needed to evaluate specific recruitment strategies and identify evidence-based approaches to enhance participation of underrepresented groups in research. A systematic review of dementia prevention trials in the United States demonstrated that studies employing community-centered approaches, such as referrals and word-of-mouth, achieved higher levels of racial and ethnic diversity compared to other recruitment strategies [9]. Other studies have shown that for individuals who are new to research, it is especially important that researchers promote trustworthiness and transparency starting with the initial study contact, as this is associated with participant receptiveness to learn about the research [10].. Effective recruitment strategies are contingent upon tailoring approaches to the specific characteristics and needs of the target population and relevant subgroups, including individuals with low socioeconomic status and limited health literacy. Such customization enhances accessibility, reduces participation barriers, and improves the representativeness of enrolled cohorts. For example, providing assistance with reading or utilizing paper surveys versus multimedia or voice assisted online surveys may be considered [11-13]. These approaches are considered standard practice and are routinely employed in population-based studies. For example, the U.S. Census Bureau offers assistance and multiple modes of survey completion, including mail, online, and inperson. [14].

A review of sampling techniques for recruiting “hard-to-reach populations” in health research found that success was not determined by the specific recruitment approach employed. Rather, successful recruitment largely depended on whether researchers accounted for *characteristics* of the target population and possessed sufficient knowledge of those characteristics. [15]. “Hard-to-reach” or “hardly reached” are terms used to refer to groups or sub-groups of populations that are not typically represented in research [16]. A scoping review evaluating the operationalization of outreach within high-risk groups noted that while the definition of hard-to-reach may vary by discipline, these groups share attributes of experiencing stigma, discrimination, distrust of researchers or providers [17]. Fisher et al., goes further to suggest the term “hardly reached” highlights the challenge stemming from the inability of interventions, healthcare systems, and researchers to effectively engage these individuals, rather than from the individuals own attributes [18].

In the context of Alzheimer’s disease and related dementia (ADRD) research, underrepresented groups often include individuals from specific racial and ethnic minority groups, low socioeconomic status, lower education levels or certain geographic location (e.g. rural) [19-21]. In a previous study of participants at the Wisconsin Alzheimer’s Disease Research Center (WADRC), researchers found that Hispanic and Asian individuals were underrepresented in the samples relative to their proportion in the broader state population [22]. Researchers have access to a variety of evidence-based recruitment strategies, encompassing both digital and non-digital modalities. The choice of method should be guided by the specific characteristics of the target population and relevant sub-group to ensure a representative sample. Digital recruitment has been widely implemented across disciplines, employing tools such as online advertisements and social media, and administering electronic consent and survey administration over the internet [23]. These approaches are commonly used to increase recruitment rates and broaden reach, as demonstrated in reviews of randomized control trial (RCTs) health research [24]. Moreover, a systematic review comparing digitally recruited and traditionally recruited (non-digitally) cohorts found that digital recruitment can effectively expand geographic reach and shorten recruitment timelines, yielding higher mean and median monthly enrollment compared with conventional methods [25]. Digital recruitment has also been used in ADRD research. Digital advertising and surveys have demonstrated successful enrollment of Black older adults, and online websites and social media platforms have also successfully recruited Latino ADRD caregivers. They also reported successfully recruiting Latino participants from diverse subgroups beyond those represented in their local region [21, 26, 27]. In addition, social media advertisements and media coverage have been used to raise awareness of, and enroll individuals into, the Alzheimer’s Prevention Registry, demonstrating the potential of digital recruitment methods for ADRD research [28].

Partnering with community health workers (CHWs) through non-digital, or in-person recruitment can be another approach to encourage research participation from “hardly reached” groups. CHWs are trusted members of their communities who serve as a vital link between the healthcare system and the people they serve [29, 30]. They share important health information and promote healthy lifestyle behaviors across a range of health issues, contributing to the reduction of health disparities [8]. CHWs routinely help individuals access healthcare services and programs and provide culturally appropriate health education [31]. Research demonstrates that CHWs help bridge gaps between healthcare providers and individuals, particularly in marginalized communities where cultural linguistic barriers are prevalent [32]. A scoping review further underscores their integral role in health intervention research, including participant recruitment, intervention implementation, and data collection [33]. CHWs have been engaged across diverse study designs including epidemiological, cross-sectional, randomized control trials, and mixed-methods, reflecting the value that researchers place on CHW collaboration [34]. Additionally, a systematic review found that that CHWs most commonly contribute to recruitment in studies focused on occupational safety and health, cancer screening, mental health, and health disparities [35-39]. Evidence consistently shows that CHW involvement enhances recruitment outcomes. For example, a randomized control trial (RCT) of Mexican–American adults with diabetes, CHW outreach yielded the highest number of participants screened and randomized [40]. Similarly, a cancer screening study that integrated CHWs into recruitment successfully enrolled 3,045 participants and showed that CHW-led navigation increased screening uptake and follow-up, highlighting the essential role of CHWs in clinical research [41].

Although both digital (online) and non-digital (in-person) recruitment—including CHW-led approaches– show promise across research areas, there remains a gap in the literature regarding the systematic evaluation of these strategies specifically within ADRD research [15, 17, 42]. A scoping review of online recruitment among older adults with probable dementia highlighted substantial gaps in knowledge regarding effective digital strategies for this population. Studies using social media reported highly variable recruitment rates, ranging from 0 to 100%, and identified unfamiliarity with technology, limited technological proficiency, and internet access as potential barriers [43]. Additionally, while ADRD researchers utilizing digital recruitment have successfully recruited traditionally underrepresented racial and ethnic groups, their samples often skew towards higher educational attainment (e.g., > 12 years of education or some college or technical school education). In light of these findings, researchers emphasize the need for future research to compare the effectiveness of different recruitment strategies and to employ a combination of communication channels to align with participant preferences and capabilities, thereby maximizing outreach and inclusivity.

Similarly, for non-digital, in-person recruitment with CHWs, many studies provide limited detail on the heterogeneous approaches used to train or support CHWs and often lack rigorous evaluation of recruitment outcomes. This gap underscoring the need for more comprehensive research to optimize the integration of CHWs in ADRD studies and to address persistent disparities in U.S. Hispanic/Latino representation [44-47]. Additionally, studies that incorporate CHW integration in ADRD studies typically describe the methods of CHW involvement without comparing outcomes to similar research without CHWs [33]. A systematic review of recruitment and retention of underrepresented populations in ADRD found that while researchers note their efforts to recruit certain groups, there is limited and inconsistent evidence on effective strategies or the details of the specific contexts of the subgroups within the target population —making it hard to generalize or replicate [19]. Similarly, a scoping review assessing NIH-sponsored research for equitable inclusions of under-represented groups and characterizing the respective study populations—further highlighted the need of future research to capture these details [20]. Our study adds to the literature by comparing two distinct recruitment strategies (digital online vs non-digital in-person CHWs) and describing the differences between participants engaged by recruitment strategy and survey outcomes. This analysis will help us identify approaches best suited for specific Hispanic/Latino population subgroups, including those with lower literacy and lower educational attainment, and may provide guidance for future studies aiming to recruit these sub-groups. Furthermore, our study also will provide information on the gaps in dementia knowledge according to specific participant characteristics, which will provide guidance to future research studies seeking to increase knowledge or awareness of ADRD in diverse populations.

## Methods

### Study Design

This study presents results from an observational cross-sectional study that conducted surveys in various cities across the United States (US) including Puerto Rico. The survey aimed to understand Hispanic/Latino perspectives and knowledge on memory problems, dementia, and Alzheimer’s disease. The study underwent an ethical review by the Institutional Review Board (IRB) at the University of Wisconsin—Madison and was determined to meet the criteria for exempt human subjects research as defined under 45 CFR 46: Category 2: Research involving the use of educational tests, surveys, interviews. This paper presents a comparative analysis of the recruitment strategies used in the study.

### Recruitment Approaches

Surveys were conducted online without CHWs or in-person by CHWs from September 2023 to April 2024, these were available in English and Spanish, and all the recruitment materials were bilingual across the study. This dual approach was intentional, as the investigators were located in Madison, WI and Los Angeles, CA, which allowed for broader geographic representation to oversample Hispanic/Latinos.

### Survey Instrument

The survey was available in English and Spanish and included a total of 54 items that took an average of 15 min to complete. The survey included demographic questions (e.g. age, gender, country of heritage, education, and experience caring for older adults or people with memory problems), and 35-items for the validated instrument titled “Beliefs affecting health seeking intentions in cognitive decline (BESIC),” [48-50]. The BESIC utilizes 5-point Likert scale questions that assess perceived likelihood of a condition or symptom to be associated with the dementia or Alzheimer’s Disease (1 = Not at all, 2 =A little, 3 = Somewhat, 4 = Very or Quite a bit, 5 = Extremely or A great deal). The instrument also included modified questions from the Cultural Beliefs about ADRD (CBAD) survey, and the Basic Knowledge in ADRD scale (BKAD) which measures knowledge about Alzheimer’s disease. The BESIC instrument demonstrated a strong internal consistency, with all sub-domains showing acceptable to good reliability (Cronbach Alpha: 0.88 for overall knowledge, 0.81 for knowledge about risk factors, and 0.82 for knowledge about prevention). Confirmatory factor analysis supported a five-factor congeneric model, and Multigroup Confirmatory Factor Analysis (MGCFA) confirmed configural and weak measurement invariance across English and Spanish versions. These findings support the use of BESIC as a reliable and culturally adaptable tool for assessing beliefs affecting health-seeking intentions in cognitive decline among Latino populations.

### Approach A: Digital Recruitment and Engagement

Survey links were distributed between September 2023 to April 2024 through a variety of channels, including email lists, social media posts, website advertisements, and printed flyers containing a QR code that directed individuals to the survey website. Recruitment emails were sent by community partners and organizations using their preferred channels and list-servs and were not asked to track these by the study team. This strategy was intentional to minimize intrusive outreach from unfamiliar senders and to leverage trusted networks. Social media recruitment followed the same approach, using personal and organizational accounts without paid advertisements or tracking tools. As a result, no digital analytic data including impressions, clicks, or engagement rates are available. Overall, we estimate that around 7,000 individuals were contacted through the digital approach. Printed flyers were distributed over a six-month period at community centers, local businesses, health fairs, and events in Milwaukee and Dane Counties. All recruitment materials were provided in both English and Spanish, using flyers that were the same used across all recruitment approaches of the study.

The online surveys were collected using Qualtrics, a secure, cloud-based survey platform that provides robust data collection and comprehensive analytics. While completing the survey, individuals selected the language of their preference and could change the language of the survey at any point. To maintain data integrity and optimize response quality, we implemented a two-phase survey process. Phase one consisted of a brief eligibility screening questionnaire with six items: two eligibility questions (age and Hispanic/Latino identity), two security questions (CAPTCHA and response replication), and two fields for contact information (name and email). The screener took approximately two minutes to complete. Eligible individuals received personalized survey links via email within three days of completing the screener. No compensation was provided for completing the screener. Data quality was monitored through analysis of submission patterns and completion times, screening of email authenticity, and Qualtrics’ built-in fraud detection metrics. Survey attempts originating outside the United States or Puerto Rico, were identified by Qualtrics through IP addresses and were unable to proceed with completion. Qualified survey participants who completed the online screening questionnaire received three email reminders over a one-month period to start or complete the survey. Participants that completed the online survey were offered assistance to complete the survey and a phone number to call should they have any inquiries. The phone line voicemail was available with the message in English and Spanish, and all the phone calls were returned within 48 h.

### Approach B: Non-digital, CHW-Led Recruitment and Engagement

In Los Angeles, the research team employed a community-focused recruitment strategy, utilizing CHWs to identify potential study participants over a one-month period between March 13, 2024, to April 22, 2024. This targeted recruitment approach aimed to promote the study within Hispanic/Latino communities that may have distrust with the medical community and research [5, 51]. The CHWs were bilingual in English and Spanish, and of similar background as the target study participants, promoting trust and rapport [10, 32]. A total of 10 CHWs were recruited by the study team through a local community organization to help facilitate the completion of a total of 309 surveys for the memory study. All CHWs were female, and reported their country of heritage as Mexico, Guatemala, and Nicaragua. The majority of CHWs reported prior experience working on research studies. CHWs represented diverse geographic areas of Los Angeles including, central, southeastern, and south regions, all areas that are heavily populated with Hispanic/Latinos. The CHWs participated in a 1-h kick-off meeting at a local community center. During the meeting, the study team provided refreshments and explained the purpose of the study. The study team reviewed the study with CHWs, distributed flyers, and provided CHWs with University of Wisconsin-Madison promotional materials valued at less than $2 per item. These giveaways consisted of pocket-sized sewing kits, reusable ice packs, and pillboxes to assist with recruitment of potential participants. At the end of the kick-off meeting, CHWs who were willing to continue assisting with study recruitment signed a memorandum of understanding of their role in the study. During the recruitment process, CHWs participated in a weekly, virtual check-in meeting where the study team provided updates on recruitment, enrollment, and survey completion. CHWs also shared their experiences and progress in recruiting participants, and they had the opportunity to raise any concerns or issues that may have arisen while working in the community that could have prompted an update to the recruitment process. Despite these discussions, no changes to the recruitment were deemed necessary and CHWs reported a high level of survey completion. CHWs were available for questions and completed a paper survey with the participants. We included CHW-identified participants who independently scanned the QR code and self-administered the online survey in the digital group. At the end of the recruitment phase, the study team held a closeout event for the CHWs, during which each CHW received a certificate of completion and a stipend for the respective number of participants they recruited. CHWs received a $175 gift card as an incentive for recruiting a goal of 10 participants each.

Completed paper surveys were entered into REDCap by two independent research staff members who each entered the same survey data separately [52]. The two sets of entries were then electronically compared by a data scientist using REDCap’s data comparison tool, which identified any discrepancies. When inconsistencies were detected, they were carefully reviewed by the data scientist, who referred to the original survey for accurate resolution. All study participants in the digital and non-digital approach received an electronic or physical gift card in the amount of $15 for completing the survey.

### Study Participants

The inclusion criteria for the study were: (a) participant self-identified as Hispanic/Latino, (b) 18 years or older and (c) living in the United States or Puerto Rico. This report presents results from 834 survey respondents, who represent 81% of the total population of the study, the remaining responses applied two additional recruitment methods that are outside of the scope of this report (mail-in surveys to a representative sample of households in Wisconsin, inperson outreach at health fairs and community events).

### Analysis

Data were summarized using descriptive statistics, which included measures of central tendency, such as means and medians, as well as measures of dispersion like standard deviations for continuous variables. For categorical variables, frequencies and percentages were reported. Preliminary analysis indicated that missing data were random; therefore, all observations were included in the analysis without any imputation. This approach ensures that the results reflect the full dataset.

For the purpose of the analysis, the recruitment approaches were the independent variables, and the outcomes of interest were demographics and knowledge about dementia. The knowledge score was derived from the modified-BKAD items within the BESIC instrument. The BKAD includes 28 items, and was scored using a methodology consistent with the approach described by Weise et al. [50]. Responses, originally on a 5-point scale, were dichotomized: values of 1 and 2 were recoded as ‘No’, and values of 3, 4, and 5 were recoded as ‘Yes’. These were then classified as correct or incorrect based on the key for the question and existing evidence. Each correct response was assigned one point, and the total score was calculated as the sum of these points, resulting in a maximum possible score of 28.

Associations between demographic variables and the two recruitment strategies were assessed using the Chisquare test. The non-parametric Mann–Whitney U test was employed BKAD scores to compare between the CHW-assisted group and the digital group, while the t-test was used for individual items of the BESIC survey. The statistical analyses were performed using SAS software, version 9.4. The assumptions for each test were verified, and a significance threshold was set at *p* < 0.05.

## Results

Of the 834 responses in this report, 526 participants (63.06%) completed the study online without CHWs, while 308 participants (36.93%) completed the survey through the CHW recruitment approach. Both recruitment strategies yielded a similar gender distribution, with about 72% of participants identifying as female. However, we found significant demographic differences between the two groups in terms of age, language, country of heritage, education, and caregiving status.

Table 1 indicates that most participants in the CHW-assisted group were aged between 26 and 64 years (75.00% vs. 54.58%, *p* < 0.001). All participants preferred Spanish as their language of communication. Additionally, all participants in this group completed the survey in Spanish (100% vs. 28.90%). This group predominantly identified as being of Mexican (45.10%) and Central American heritage (33.66%). Furthermore, a higher percentage of participants in the CHW-assisted group had less than a high school diploma compared to those in the digital group (29.14% vs. 5.13%, *p* < 0.001). More participants in the CHW-assisted group indicated that they did not care for anyone aged 60 years or older (81.58% vs. 69.37%, *p* < 0.001) and were uncertain about providing care for someone with dementia (13.44% vs. 10.85%, *p* < 0.001) compared to those in the digital group.

In contrast, most respondents in the digital group completed the survey in English (71.10% vs. 0%, *p* < 0.001) and had attended some college or obtained a higher level of education (78.69% vs. 28.14%, *p* < 0.0001) compared to the CHW-recruited group. A larger percentage of participants in this group identified as Puerto Rican (16.60% vs. 0.65%) and South American (11.45% vs. 2.29%) compared to the CHW-assisted group. Additionally, a greater percentage of the digital group reported caring for someone aged 60 or older (30.63% vs. 18.42%, *p* < 0.05) and someone with dementia (64.69% vs. 27.87%, *p* < 0.001). Overall, the digital group had higher levels of education, greater diversity in participants’ countries of heritage, and reported more engagement in caregiving for older adults and individuals with dementia.

Table 2 presents the results from multiple independent t-tests, highlighting significant differences in the knowledge about Alzheimer’s disease and dementia, and beliefs about risk factors and prevention strategies between participants according to the two recruitment strategies used.

Participants recruited without the assistance of CHWs demonstrated a better understanding of Alzheimer’s disease, specifically regarding risk factors and the importance of preventive measures. In contrast, those recruited through CHWs showed several misconceptions about the disease and its prevention, indicating notable differences in knowledge and beliefs about Alzheimer’s disease between the two groups. When analyzing these differences using the BKAD score, participants in the CHW-assisted group had slightly lower knowledge about Alzheimer’s Disease compared to the digital group, with averages of 19.89 points versus 21.85 points out of 28. This difference was statistically significant (*p* < 0.001), suggesting that participants in the CHW-assisted group had a relatively less comprehensive understanding of Alzheimer’s disease.

## Discussion

Findings from this study indicate that digital recruitment strategies were associated with recruiting a population with higher educational levels, which correlates with enhanced knowledge and awareness of Alzheimer’s disease and other memory disorders [53-56]. Conversely, recruitment facilitated by CHWs was associated with recruiting a greater proportion of Spanish-preferring participants, and those with lower educational attainment. These insights underscore the need to tailor recruitment and educational strategies to the distinct needs of different demographic groups, informing experimental work to advance recruitment science and enhance inclusivity and effectiveness in health education and research [57, 58]. Our findings add to existing literature by showing that recruitment strategies are associated with participant demographics and with survey responses on knowledge and beliefs about memory disorders.

Our findings are consistent with existing literature demonstrating that CHWs can engage groups that are traditionally under-represented in research. For instance, all participants in the CHW-assisted group were Spanish-preferring participants and most had at least a high school education or less, compared to those in the digital group, who had attended some college or more. The involvement of CHWs in the recruitment process had several advantages. They were able to bridge a gap between the research team and individuals with lower educational attainment and who preferred Spanish as their spoken language, a group that the digital survey approach did not reach. As a result, our study demonstrated that sub-groups within the broader target population—such as distinct Hispanic/Latino communities—can differ in their of levels of knowledge and in the types or prevelence of misconceptions about health conditions like Alzheimer’s disease. Since the digital survey approach did not recruit individuals with lower educational attainment, our findings highlights the necessity of deploying diverse recruitment strategies tailored to reach specific target groups [11-13, 15]. This aligns with prior evidence that CHWs, as trusted community members, effectively bridge communities and the healthcare sector—including research—and excel at recruiting underrepresented groups whose knowledge and experiences are often underrecognized. [29, 30, 59]. [32]. [17].

Our results showed that the differences in demographics of the participants were associated with differences in knowledge about ADRD. Individuals recruited through CHW-assisted methods showed less accurate knowledge about Alzheimer’s disease and held more misconceptions, particularly the belief that Alzheimer’s is a normal part of aging, which has been previously reported for U.S. Hispanic/Latino communities [60]. These misconceptions may reduce the likelihood of this population recognizing early symptoms and seeking timely medical assistance, which may increase the risk of delayed diagnosis and treatment.

The digital approach recruited more Latino participants who had caregiving experience for older adults with dementia, representing two important subgroups to engage in ADRD research. This is particularly notable as engaging caregivers may facilitate the participation of the older adults they care for, potentially broadening access to ADRD research opportunities. This is also important as ADRD studies often exclude adults younger than 65 years [61]. Caregivers may also have a family history of ADRD, which could further motivate involvement and inform study findings [62]. However, we acknowledge that the survey was not designed to determine whether caregiving experience was for a biological family member with dementia or for someone else, limiting our conclusions about caregiver roles in this study.

Our results also coincide with others that report that significant knowledge gaps exist among U.S. Hispanic/Latino communities regarding the causes, prevention, and management of Alzheimer’s disease, with many attributing it to stress or fate [63]., rather than recognizing modifiable risk factors and the potential benefits of preventive behaviors like exercise [64, 65]. The lack of awareness and knowledge is compounded by barriers such as limited access to culturally and linguistically appropriate educational materials, particularly for older adults who may not use the internet or have low health literacy. Collectively, these findings underscore the urgent need for culturally appropriate educational interventions to improve awareness and prevention of memory-related disorders in Hispanic/Latino and lower educational attainment.

Regarding approach intensity and cost, evidence suggests that online recruitment can be cost-effective and enhance geographic diversity, a potential strength of online recruitment that could enhance representativeness relative to the broader U.S [66]. Our study did not systematically track or analyze the financial costs associated with each method in this study, as online recruitment was conducted through a combination of in-kind support from community partners, volunteer efforts, and institutional resources, making it difficult to estimate direct costs. We did not record the geographic location of participants. Future studies should collect geographic data to evaluate and leverage the representativeness of online samples. However, studies have shown that digital approaches tend to underrepresent individuals from lower socioeconomic and lower literacy backgrounds—two factors that are often correlated. This is especially relevant for Latino populations, where approximately 54% have a high school education or less [67]. As such, exclusive reliance on online recruitment may inadvertently exacerbate gaps in research participation. These considerations underscore the importance of combining digital strategies with community-based approaches, such as engagement with CHWs, to ensure more equitable and representative recruitment.

Despite the insights gained from this study, several important limitations need to be considered when interpreting these results. First, the study utilized a non-randomized design, which limits the ability to infer causality. This makes it challenging to determine whether the differences in knowledge and beliefs were primarily due to the recruitment method or other unmeasured pre-existing demographic characteristics. However, we believe that this limitation is alleviated by the numerous studies that have demonstrated that the recruitment method itself is a significant factor influencing who participates in research studies, frequently outweighing the role of demographic or clinical characteristics. Finally, the study relied on self-reported survey data, which can be affected by biases such as social desirability and recall bias. For example, participants in the CHW group completed the survey on paper, while those in the digital group answered it via an online survey. Participants in the CHW group might have responded in a way they thought would be more favorable to the CHW administering the survey. Additionally, our CHW-led recruitment was conducted in Los Angeles, CA, a geographical region with a greater density of Latino population, whereas the online survey was broadly shared within the US, including Puerto Rico. As such, the observed differences may also reflect local sociodemographic or regional context, limiting generalizability. Future research should evaluate both recruitment strategies across multiple regions with varying density and socioeconomic contexts to assess the consistency of these observed associations.

Another limitation of this study is the absence of detailed data on the number of prospective participants approached by CHWs, as well as the number who declined participation and their reasons for doing so. Collecting such data could have provided deeper insights into the recruitment process and potential selection bias. However, collecting this information was not feasible due to logistical considerations. Additionally, while this study does not provide a monthly analysis comparing survey completion between the CHW-led and digitally recruited groups as the goal of the study was to demonstrate that comprehensive recruitment strategies are needed to achieve diversity, future studies should consider conducting sub-analysis exploring the impact of survey administration methods and consider the impact of external factors such as timing of email distributions, community events and presentations where study flyers might be shared [68]. Furthermore, for the digital approach, social media engagement metrics such as likes, shares, comments, and follower counts were not collected, as these were beyond the scope of this study. Future research comparing participant engagement through different digital and non-digital approaches should measure these to describe and better understand prospective study participant engagement. [69]. Despite these limitations, it is noteworthy that CHWs reported a high level of survey completion among those approached, indicating substantial engagement and interest from potential participants. Nevertheless, without specific data, we are unable to quantitatively assess engagement levels or elucidate the reasons for participant refusal. Future research endeavors could address this gap by incorporating comprehensive recruitment metrics, thereby enhancing the understanding of participant engagement and refusal dynamics. Additionally, to build on our current findings, future research could explore the expansion of the CHW approach to different geographic regions to assess the consistency and generalizability of the results. Such studies could provide insights into regional variations and the adaptability of the CHW model across diverse settings. Examining how the level of acculturation among participants influences their interactions with CHWs and their engagement with health interventions could offer valuable insights. Exploring this relationship could help tailor health strategies to meet the needs of culturally diverse populations.

Future research should continue to explore and refine partnerships with CHWs. These individuals have established trust and rapport within their communities, making them valuable liaisons for disseminating tailored information about Alzheimer’s disease. Engaging CHWs in the co-creation of culturally sensitive educational materials and interventions could enhance recruitment, improve participant engagement and retention, and support the design of interventions aimed at increasing ADRD awareness knowledge of prevention strategies. By improving awareness and understanding, these efforts can empower individuals to seek early care, ultimately leading to better outcomes for both patients and their families. Additionally, future studies should explore how involving CHWs, and culturally tailored outreach can be adapted for clinical trials or research protocols that require greater participant commitment, such as brain imaging, biospecimen collection, or longitudinal follow-up. Understanding how to translate these approaches to higher-burden studies will be critical for improving representation and engagement in dementia research and, ultimately, to accelerate the generation of generalizable findings that inform prevention and care for diverse populations.

## Figures and Tables

**Table 1:** Comparison of demographic characteristics between CHW and digitally recruited participants (*N* = 834)

	CHW Recruited (*n* = 308)	Digitally Recruited (*n* = 526)	*p* value
Age			
Average Age (SD)	45.68 (15.49)	48.07 (18.26)	
18–25 years	39 (12.83%)	128 (24.62%)	< 0.001
26–64 years	228 (75.00%)	274 (54.58%)	
= > 65 years	37 (12.17%)	118 (22.69%)	
Gender			
Man	86 (28.38%)	129 (25.44%)	0.11
Woman	217 (71.62%)	372 (73.37%)	
Language			
Spanish	308 (100%)	152 (28.90%)	< 0.001
English	-	374 (71.10%)	
Country or Region of Heritage			
Caribbean	-	27 (5.15%)	< 0.001
Centro America	103 (33.66%)	23 (4.39%)	
Mexico/Chicano	138 (45.10%)	195 (37.21%)	
Puerto Rico	2 (0.65%)	87 (16.60%)	
South America	7 (2.29%)	60 (11.45%)	
Unspecified	56 (18.30%)	132 (25.19%)	
Education			
No formal education	8 (2.65%)	3 (0.59%)	< 0.001
Elementary School	51 (16.89%)	12 (2.37%)	
Middle School	29 (9.60%)	11 (2.17%)	
High School	91 (30.13%)	58 (11.44%)	
Technical or Trade	38 (12.58%)	24 (4.73%)	
School			
Some College	39 (12.91%)	107 (21.10%)	
College or more	46 (15.23%)	292 (57.59%)	
Provided care for			
60 years or older			
No	248 (81.58%)	351 (69.37%)	0.002
Yes	56 (18.42%)	155 (30.63%)	
Provided care for some-one with dementia			
No	179 (58.69%)	124 (24.46%)	< 0.001
Yes	85 (27.87%)	328 (64.69%)	
Unsure	41 (13.44%)	55 (10.85%)	

Subtotals may not sum to the total *N* due to missing data.

**Table 2:** Average BESIC scores for individual items by CHW and digitally recruited participants (Scale: 1 = Not at all, 5 = Extremely or a great deal)

	CHW Recruited (*n* = 308)	Digitally Recruited (*n* = 526)	p value
How normal is it to forget things as part of growing older?	3.14 ± 0.99	3.13 ± 0.90	0.85
How normal is Alzheimer’s disease as a part of growing older?	2.96 ± 1.09	2.12 ± 1.04	**< 0.001**
How likely is it that there are things you can do to reduce your risk of getting Alzheimer’s disease?	3.07 ± 1.11	3.31 ± 1.05	**0.002**
How likely is it that Alzheimer’s disease can be cured?	2.24 ± 1.17	2.10 ± 1.14	0.09
How likely are people with Alzheimer’s disease to be depressed?	3.41 ± 1.08	3.57 ± 0.99	**0.03**
How likely is it that people with Alzheimer’s disease will always be able to recognize their friends and family members?	2.28 ± 1.07	1.96 ± 0.93	**< 0.001**
Domain Knowledge – Likelihood			
How likely is someone to have Alzheimer’s disease if…			
…they stop taking part in social activities?	3.15 ± 1.24	3.01 ± 1.11	0.08
…they wear a heavy coat when it is hot outside?	2.63 ± 1.32	2.47 ± 1.25	0.08
…they forget names of familiar objects?	3.65 ± 1.09	3.64 ± 0.99	0.91
…they often forget appointments?	**3.71 ± 1.06**	**3.44 ± 1.03**	**0.0003**
…they have difficulty remembering the rules of a game they have played many times before?	3.59 ± 1.17	3.57 ± 1.03	0.85
…they have trouble following directions?	3.48 ± 1.16	3.50 ± 1.04	0.75
…they ask someone the same question over and over again?	**3.79 ± 1.08**	**3.98 ± 0.94**	**0.006**
…they have trouble counting money?	3.46 ± 1.17	3.54 ± 1.09	0.28
…they lose their keys from time to time?	**3.56 ± 1.26**	**2.79 ± 1.26**	**< 0.001**
Domain Knowledge—Risk			
How much is someone’s risk of getting Alzheimer’s disease increased if…			
…a parent had it?	**3.20 ± 1.38**	**3.63 ± 1.01**	**< 0.001**
…they have a serious hit to their head?	2.64 ± 1.29	2.63 ± 1.16	0.95
…they have high blood sugar?	2.43 ± 1.25	2.58 ± 1.12	0.09
…they have high blood pressure?	2.46 ± 1.24	2.56 ± 1.09	0.21
…they have had a stroke?	2.91 ± 1.36	2.86 ± 1.20	0.63
…they have poor nutrition?	**2.61 ± 1.27**	**2.87 ± 1.16**	**0.002**
…they do not get enough sleep?	2.97 ± 1.24	3.08 ± 1.11	0.21
…they do not take care of themselves?	3.03 ± 1.29	3.15 ± 1.16	0.14
…they experienced a difficult life or traumatic event?	2.86 ± 1.30	2.80 ± 1.21	0.58
Domain Knowledge—Prevention			
To prevent or slow Alzheimer’s disease, how important is it to…			
…get help with dementia symptoms early?	3.99 ± 1.15	4.41 ± 0.81	**< 0.001**
…stay mentally active?	4.17 ± 0.97	4.52 ± 0.70	**< 0.001**
…be with others to keep the memory sharp?	3.84 ± 1.09	4.08 ± 0.92	**0.001**
…have your memory tested regularly if you are over 65?	3.93 ± 1.08	4.10 ± 0.94	**0.02**
…stay physically active?	4.06 ± 0.98	4.26 ± 0.80	**0.001**
…keep written lists as reminders?*	3.59 ± 1.20	3.34 ± 1.16	**0.004**
…try very hard to remember things?*	3.49 ± 1.16	3.07 ± 1.23	**< 0.001**
…take care of high blood pressure?	3.62 ± 1.20	3.75 ± 1.12	0.11

* These reflect misconceptions about memory problems, and compensatory mechanisms

## Data Availability

Datasets used and/or analyzed during the current study are available from the corresponding author upon reasonable request.

## References

[1] National Academies of Sciences E, Medicine, Policy, Global A, Committee on Women in Science E, Medicine, The National Academies Collection: Reports funded by National Institutes of Health. In: Bibbins-Domingo K, Helman A, editor(s). Improving Representation in Clinical Trials and Research: Building Research Equity for Women and Underrepresented Groups. Washington (DC): National Academies Press (US) Copyright 2022 by the National Academy of Sciences. All rights reserved. 2022. 36137057

[2] U.S. Food & Drug Administration. Drug Trials Snapshots Summary Report 2023. Place. 2023.

[3] Bonevski B, Randell M, Paul C, Chapman K, Twyman L, Bryant J, Reaching the hard-to-reach: a systematic review of strategies for improving health and medical research with socially disadvantaged groups. BMC Med Res Methodol. 2014;14(1):42. 10.1186/1471-2288-14-42. 24669751 PMC3974746

[4] Erves JC, Mayo-Gamble TL, Malin-Fair A, Boyer A, Joosten Y, Vaughn YC, Needs, priorities, and recommendations for engaging underrepresented populations in clinical research: a community perspective. J Community Health. 2017;42(3):472–80. 10.1007/s10900-016-0279-2. 27812847 PMC5408035

[5] Escarce JJ, Kapur K. Access to and quality of health care. In: F TMM, editor(s). Hispanics and the future of America. Washington (DC): National Academies Press (US); 2006. pp. 410–78.

[6] Hussain-Gambles M, Atkin K, Leese B. Why ethnic minority groups are under-represented in clinical trials: a review of the literature. Health Soc Care Community. 2004;12(5):382–8. 10.1111/j.1365-2524.2004.00507.x. 15373816

[7] National Academies of Sciences E, and Medicine. Why Diverse Representation in Clinical Research Matters and the Current State of Representation within the Clinical Research Ecosystem. In: Bibbins-Domingo K, Helman A, editor(s). Improving Representation in Clinical Trials and Research: Building Research Equity for Women and Underrepresented Groups. Washington (DC); 2022. 36137057

[8] Washington V, Franklin JB, Huang ES, Mega JL, Abernethy AP. Diversity, equity, and inclusion in clinical research: a path toward precision health for everyone. Clin Pharmacol Ther. 2023;113(3):575–84. 10.1002/cpt.2804. 36423203

[9] Lazaar N, Van Beek SE, Rirash AF, Papma JM, Perales-Puchalt J, Shaw AR, Diversity in United States dementia prevention trials: an updated systematic review of eligibility criteria and recruitment strategies. Dement Geriatr Cogn Disord. 2025. 10.1159/000543905. PMC12366532 39947158

[10] Behringer-Massera S, Browne T, George G, Duran S, Cherrington A, McKee MD. Facilitators and barriers to successful recruitment into a large comparative effectiveness trial: a qualitative study. J Comp Eff Res. 2019;8(10):815–26. 10.2217/cer-2019-0010. 31368793 PMC7333580

[11] Emery LF, Silverman DM, Carey RM. Conducting research with people in lower-socioeconomic-status contexts. Adv Methods Pract Psychol Sci. 2023;6(4):25152459231193044. 10.1177/25152459231193044.

[12] Theunissen MCM, Koks-Leensen MCJ, van Geenen J, Leusink GL, Naaldenberg J, Bevelander KE. Engaging underrepresented populations in public health monitoring: strategies for people with mild intellectual disability or low literacy skills. Int J Equity Health. 2025;24(1):204. 10.1186/s12939-025-02578-0. 40660218 PMC12257827

[13] Tieu L, Hobbs A, Sarkar U, Nacev EC, Lyles CR. Adapting patient experience data collection processes for lower literacy patient populations using tablets at the point of care. Med Care. 2019;57(6):140–8. 10.1097/Mlr.0000000000001030. PMC6527129 31095053

[14] Bureau USC. U.S. Census Bureau: Other Ways to Respond. In: Book/Database title. 2025. https://www.census.gov/programs-surveys/acs/respond/other-ways-to-respond.html?utm_source=chatgpt.com. Accessed October 20, 2025.

[15] Shaghaghi A, Bhopal RS, Sheikh A. Approaches to recruiting “hard-to-reach” populations into re-search: a review of the literature. Health Promot Perspect. 2011;1(2):86–94. 10.5681/hpp.2011.009. 24688904 PMC3963617

[16] Freimuth VS, Mettger W. Is there a hard-to-reach audience? Public Health Rep. 1990;105(3):232–8. 2113680 PMC1580016

[17] Jiao S, Slemon A, Guta A, Bungay V. Exploring the conceptualization, operationalization, implementation, and measurement of outreach in community settings with hard-to-reach and hidden populations: a scoping review. Soc Sci Med. 2022. 10.1016/j.socscimed.2022.115232. 35964472

[18] Fisher EB, Coufal MM, Parada H, Robinette JB, Tang PY, Urlaub DM, Peer support in health care and prevention: cultural, organizational, and dissemination issues. Annu Rev Public Health. 2014;35:363–83. 10.1146/annurev-publhealth-032013-182450. 24387085

[19] Gilmore-Bykovskyi AL, Jin Y, Gleason C, Flowers-Benton S, Block LM, Dilworth-Anderson P, Recruitment and retention of underrepresented populations in Alzheimer’s disease research: a systematic review. Alzheimer’s & Dementia: Translational Research & Clinical Interventions. 2019;5:751–70. 10.1016/j.trci.2019.09.018. 31921966 PMC6944728

[20] Godbole N, Kwon SC, Beasley JM, Roberts T, Kranick J, Smilowitz J, Assessing equitable inclusion of underrepresented older adults in Alzheimer’s disease, related cognitive disorders, and aging-related research: a scoping review. Gerontologist. 2023;63(6):1067–77. 10.1093/geront/gnac060. 35472166 PMC10353042

[21] Miller MJ, Diaz A, Conti C, Albala B, Flenniken D, Fockler J, The ADNI4 digital study: a novel approach to recruitment, screening, and assessment of participants for AD clinical research. Alzheimers Dement. 2024;20(10):7232. 10.1002/alz.14234. 39219153 PMC11485063

[22] Ma Y, Mora Pinzon MC, Buckingham WR, Bersch AJ, Powell WR, LeCaire TJ, Comparison of sample characteristics of Wisconsin Alzheimer’s Disease research center participants with the Wisconsin state population-an evaluation of the recruitment effort. Alzheimer’s Dementia: Transl Res Clin Interv. 2025;11(1):e70036. 10.1002/trc2.70036. PMC11736623 39822591

[23] Gupta A, Calfas KJ, Marshall SJ, Robinson TN, Rock CL, Huang JS, Clinical trial management of participant recruitment, enrollment, engagement, and retention in the SMART study using a marketing and information technology (MARKIT) model. Contemp Clin Trials. 2015;42:185–95. 10.1016/j.cct.2015.04.002. 25866383 PMC4450113

[24] Frampton GK, Shepherd J, Pickett K, Griffiths G, Wyatt JC. Digital tools for the recruitment and retention of participants in randomised controlled trials: a systematic map. Trials. 2020;21(1):478. 10.1186/s13063-020-04358-3. 32498690 PMC7273688

[25] Moseson H, Kumar S, Juusola JL. Comparison of study samples recruited with virtual versus traditional recruitment methods. Contemp Clin Trials Commun. 2020;19:100590. 10.1016/j.conctc.2020.100590. 32637722 PMC7327265

[26] Quinones MM, Silva C, Ross C, Sorensen S, Serrano R, Van Orden K, Recruiting socially disconnected Latinos caring for a person with Alzheimer’s disease and related dementias during the COVID-19 pandemic: lessons learned. Clin Gerontol. 2023. 10.1080/07317115.2023.2197895. PMC10542654 37005703

[27] Kim JE, Knox M, Grill JD, Witbracht M, Zhang Y, Salazar H, Virtual data collection strategies in research on Alzheimer’s disease and related dementias (ADRD). Innov Aging. 2025;9(5):igaf026. 10.1093/geroni/igaf026. 40454424 PMC12123064

[28] Langbaum JB, High N, Nichols J, Kettenhoven C, Reiman EM, Tariot PN. The Alzheimer’s prevention registry: a large internet-based participant recruitment registry to accelerate referrals to Alzheimer’s-focused studies. J Prev Alzheimers Dis. 2020;7(4):242–50. 10.14283/jpad.2020.31. 32920626 PMC7534299

[29] Association APH. Community Health Workers. In: Book/Database title. American Public Health Association. n.d. Accessed 03/03/25. https://www.apha.org/apha-communities/member-sections/community-health-workers

[30] Olaniran A, Smith H, Unkels R, Bar-Zeev S, van den Broek N. Who is a community health worker? - a systematic review of definitions. Glob Health Action. 2017;10(1):1272223. 10.1080/16549716.2017.1272223. 28222653 PMC5328349

[31] Centers for Disease Control and Prevention (U.S.). National Center for Chronic Disease Prevention and Health Promotion. The role of community health workers in addressing food and nutrition security and social support. Atlanta (GA): CDC Division of Diabetes Translation. 2022.

[32] Shahidi H, Sickora C, Clancy S, Nagurka R. Community health workers recruitment from within: an inner-city neighborhood-driven framework. BMC Res Notes. 2015;8(1):715. 10.1186/s13104-015-1700-0. 26602537 PMC4658807

[33] Coulter K, Ingram M, McClelland DJ, Lohr A. Positionality of community health workers on health intervention research teams: a scoping review. Front Public Health. 2020. 10.3389/fpubh.2020.00208. PMC7308474 32612967

[34] Nebeker C, Giacinto RE, Pacheco BA, López-Arenas A, Kalichman M. Prioritizing competencies for “research” promotores and community health workers. Health Promot Pract. 2021;22(4):512. 10.1177/1524839920913548. 32228241

[35] Baezconde-Garbanati L MB, Ochoa C, Optimizing Engagement of the Latino Community in Cancer Research. In: RAT EJ, editor(s). dvancing the Science of Cancer in Latinos: Building Collaboration for Action. Cham (CH): Springer. 2022. 37816103

[36] Brown LD, Vasquez D, Salinas JJ, Tang X, Balcazar H. Evaluation of healthy fit: a community health worker model to address Hispanic health disparities. Prev Chronic Dis. 2018;15:E49. 10.5888/pcd15.170347. 29704370 PMC5951157

[37] Jimenez DE, Reynolds CF 3rd, Alegria M, Harvey P, Bartels SJ. The happy older Latinos are active (HOLA) health promotion and prevention study: study protocol for a pilot randomized controlled trial. Trials. 2015;16:579. 10.1186/s13063-015-1113-3. 26683695 PMC4683729

[38] Savas LS, Atkinson JS, Figueroa-Solis E, Valdes A, Morales P, Castle PE, A lay health worker intervention to improve breast and cervical cancer screening among Latinas in El Paso, Texas: a randomized control trial. Prev Med. 2021;145:106446. 10.1016/j.ypmed.2021.106446. 33548363

[39] Swanberg JE, Nichols HM, Clouser JM, Check P, Edwards L, Bush AM, Correction to: A systematic review of community health workers’ role in occupational safety and health research. J Immigr Minor Health. 2018;20(6):1532. 10.1007/s10903-018-0739-0. 29616387

[40] Martin MA, Swider SM, Olinger T, Avery E, Lynas CMT, Carlson K, Recruitment of Mexican American Adults for an Intensive Diabetes Intervention Trial. Ethnic Dis. 2011;21(1):7–12. PMC3704315 21462723

[41] Mojica CM, Almatkyzy G, Morales-Campos D. A cancer education-plus-navigation intervention implemented within a federally qualified health center and community-based settings. J Cancer Educ. 2021;36(1):152. 10.1007/s13187-019-01611-5. 31463809

[42] Salazar CR, Tallakson M, Corona MG, Duran E, Russ E, Hoang D, Community recruitment of underrepresented populations to the AHEAD 3-45 preclinical AD trial using novel partnerships with nursing and community-based organizations: lessons and outcomes. Alzheimers Dement. 2024;20(10):7160–73. 10.1002/alz.14211. 39210635 PMC11485091

[43] Min D, Yun JY, Parslow C, Jajodia A, Han HR. Online-based recruitment methods for community-dwelling older adults: scoping review and lessons learned from the PLAN trial. J Med Internet Res. 2025;27:e55082. 10.2196/55082. 39998873 PMC11897674

[44] Dabiri S, Raman R, Grooms J, Molina-Henry D. Examining the role of community engagement in enhancing the participation of racial and ethnic minoritized communities in Alzheimer’s disease clinical trials; a rapid review. J Prev Alzheimers Dis. 2024;11(6):1647–72. 10.14283/jpad.2024.149. 39559877 PMC11573826

[45] Gelman CR. Learning from recruitment challenges: barriers to diagnosis, treatment, and research participation for Latinos with symptoms of Alzheimer’s disease. J Gerontol Soc Work. 2010;53(1):94–113. 10.1080/01634370903361847. 20029704 PMC4049338

[46] Latinos and Alzheimer’s Disease: New Numbers Behind the Crisis. Place: USC Edward R. Roybal Institute on Aging and the Latinos Against Alzheimer’s Network. 2016. https://roybal.usc.edu/wp-content/uploads/2016/10/Latinos-and-AD_USC_UsA2-Impact-Report.pdf

[47] Raman R, Aisen PS, Carillo MC, Detke M, Grill JD, Okonkwo OC, Tackling a major deficiency of diversity in Alzheimer’s disease therapeutic trials: an CTAD task force report. J Prev Alzheimers Dis. 2022;9(3):388–92. 10.14283/jpad.2022.50. 35841239 PMC9098373

[48] Mora Pinzon M, Sayegh P, de Fernanz Cordova S, Brown R, Barrett B. Development and psychometric evaluation of a bilingual instrument for assessing beliefs affecting health-seeking intentions in cognitive decline among Latino populations. medRxiv. 2025:2025.04.24.25326381. 10.1101/2025.04.24.25326381.

[49] Wiese LK, Williams CL, Tappen RM, Newman D. An updated measure for investigating basic knowledge of Alzheimer’s disease in underserved rural settings. Aging Ment Health. 2020;24(8):1348–55. 10.1080/13607863.2019.1584880. 30869990 PMC8474126

[50] Withers M, Sayegh P, Rodriguez-Agudelo Y, Ernstrom K, Raman R, Montoya L, A mixed-methods study of cultural beliefs about dementia and genetic testing among Mexicans and Mexican-Americans at-risk for autosomal dominant Alzheimer’s disease. J Genet Couns. 2019;28(5):921–32. 10.1002/jgc4.1133. 31207006 PMC7500864

[51] Armstrong K, Ravenell KL, McMurphy S, Putt M. Racial/ethnic differences in physician distrust in the United States. Am J Public Health. 2007;97(7):1283–9. 10.2105/ajph.2005.080762. 17538069 PMC1913079

[52] Harris PA, Taylor R, Thielke R, Payne J, Gonzalez N, Conde JG. Research electronic data capture (REDCap)—a metadata-driven methodology and workflow process for providing translational research informatics support. J Biomed Inform. 2009;42(2):377–81. 10.1016/j.jbi.2008.08.010. 18929686 PMC2700030

[53] American Psychological Association (APA). Education and Socioeconomic Status. In: Book/Database title. 2017. Accessed 2/28/25. https://www.apa.org/pi/ses/resources/publications/education

[54] The Relationship between Socioeconomic Status and Literacy: How Literacy is Influenced by and Influences SES. In: Book/Database title. 2023. Accessed 02/26/25. https://sites.lsa.umich.edu/mje/2023/01/05/the-relationship-between-socioeconomic-status-and-literacy-how-literacy-is-influenced-by-and-influences-ses/

[55] Nafilyan V, Eley S, Courtin E. Differences in dementia prevention knowledge by educational attainment: an analysis of a household survey from Great Britain. BMJ Public Health. 2024;2(2):e001479. 10.1136/bmjph-2024-001479. 40018601 PMC11816588

[56] Workman J. Inequality begets inequality: income inequality and socioeconomic achievement gradients across the United States. Soc Sci Res. 2022;107:102744. 10.1016/j.ssresearch.2022.102744. 36058607

[57] Friis K, Lasgaard M, Rowlands G, Osborne RH, Maindal HT. Health literacy mediates the relationship between educational attainment and health behavior: a Danish population-based study. J Health Commun. 2016;21(sup2):54–60. 10.1080/10810730.2016.1201175. 27668691

[58] Sudhakar S, Aebi ME, Burant CJ, Wilson B, Wenk J, Briggs FBS, Health literacy and education level correlates of participation and outcome in a remotely delivered epilepsy self-management program. Epilepsy Behav. 2020;107:107026. 10.1016/j.yebeh.2020.107026. 32249034 PMC7242156

[59] National Heart L, and Blood Institute. Community Health Workers. In: Book/Database title. National Heart, Lung, and Blood Institute. 2025. https://www.nhlbi.nih.gov/education/heart-truth/CHW. Accessed 2025-03-31.

[60] Ortiz F, Fitten LJ. Barriers to healthcare access for cognitively impaired older Hispanics. Alzheimer Dis Assoc Disord. 2000;14(3):141–50. 10.1097/00002093-200007000-00005. 10994655

[61] Mitchell AK, Ehrenkranz R, Franzen S, Han SH, Shakur M, McGowan M, Analysis of eligibility criteria in Alzheimer’s and related dementias clinical trials. Sci Rep. 2024;14(1):15036. 10.1038/s41598-024-65767-x. 38951633 PMC11217383

[62] Miranda A, Rubovits E, Mudar R, Leung V, Raj M. Where are caregivers in the clinical trial? Evaluation of caregiver responsibilities in Alzheimer’s disease and related dementias clinical trials. Alzheimer Dis Assoc Disord. 2023;19(11):5316–22. 10.1002/alz.13418. 37594028

[63] Light SW, Tomasino F, Wescott A, Bernstein Sideman A, Vela A, Possin KL, Penedo FJ, Wolf MS. Perceptions, beliefs, attitudes, and knowledge of US Latino adults pertaining to dementia and brain health: a systematic review. Aging Ment Health. 2024 Mar-Apr;28(3):396–407. https://doi.org/10.1080/13607863.2023.2268050 10.1080/13607863.2023.2268050 37874117 PMC10983845

[64] Cabrera LY, Kelly P, Vega IE. Correction to: Knowledge and attitudes of two Latino groups about Alzheimer disease: a qualitative study. J Cross Cult Gerontol. 2023;38(2):221. 10.1007/s10823-023-09478-2. 37017804 PMC10238290

[65] Roberts JS, McLaughlin SJ, Connell CM. Public beliefs and knowledge about risk and protective factors for Alzheimer’s disease. Alzheimers Dement. 2014;10(5 Suppl):S381–9. 10.1016/j.jalz.2013.07.001. 24630852 PMC4163539

[66] Tsaltskan V, Sanchez Baez R, Firestein GS. Cost-effectiveness of social media advertising as a recruitment tool: a systematic review and meta-analysis. J Clin Transl Sci. 2023;7(1):e180. 10.1017/cts.2023.596. 37745929 PMC10514690

[67] Center PR. U.S. Hispanics’ Educational Attainment. In: Book/Database title. 2023. Accessed 03/03/25. https://www.pewresearch.org/chart/us-hispanics-education/

[68] Quinn SC, Butler J 3rd, Fryer CS, Garza MA, Kim KH, Ryan C, Attributes of researchers and their strategies to recruit minority populations: results of a national survey. Contemp Clin Trials. 2012;33(6):1231–7. 10.1016/j.cct.2012.06.011. 22771575 PMC3468713

[69] Mora Pinzon M, Hills O, Levy G, James TT, Benitez A, Lawrence S, Implementation of a social media strategy for public health promotion in Black, American Indian or Alaska Native, and Hispanic or Latino communities during the COVID-19 pandemic: cross-sectional study. J Med Internet Res. 2024;26:e58581. 10.2196/58581. 39657164 PMC11668992

